# A Simplified Linearized Lattice Boltzmann Method for Acoustic Propagation Simulation

**DOI:** 10.3390/e24111622

**Published:** 2022-11-08

**Authors:** Qiaochu Song, Rongqian Chen, Shuqi Cao, Jinhua Lou, Ningyu Zhan, Yancheng You

**Affiliations:** School of Aerospace Engineering, Xiamen University, Xiamen 361005, China

**Keywords:** simplified linearized lattice Boltzmann method, immersed boundary method, computational aeroacoustics

## Abstract

A simplified linearized lattice Boltzmann method (SLLBM) suitable for the simulation of acoustic waves propagation in fluids was proposed herein. Through Chapman–Enskog expansion analysis, the linearized lattice Boltzmann equation (LLBE) was first recovered to linearized macroscopic equations. Then, using the fractional-step calculation technique, the solution of these linearized equations was divided into two steps: a predictor step and corrector step. Next, the evolution of the perturbation distribution function was transformed into the evolution of the perturbation equilibrium distribution function using second-order interpolation approximation of the latter at other positions and times to represent the nonequilibrium part of the former; additionally, the calculation formulas of SLLBM were deduced. SLLBM inherits the advantages of the linearized lattice Boltzmann method (LLBM), calculating acoustic disturbance and the mean flow separately so that macroscopic variables of the mean flow do not affect the calculation of acoustic disturbance. At the same time, it has other advantages: the calculation process is simpler, and the cost of computing memory is reduced. In addition, to simulate the acoustic scattering problem caused by the acoustic waves encountering objects, the immersed boundary method (IBM) and SLLBM were further combined so that the method can simulate the influence of complex geometries. Several cases were used to validate the feasibility of SLLBM for simulation of acoustic wave propagation under the mean flow.

## 1. Introduction

The phenomenon of acoustic waves propagating in complex flows such as shear layers or a vortex often exists in aerospace engineering [1,2,3,4]. Studies have shown that such flow structures will change the characteristics of acoustic wave propagation, leading to refraction, reflection, and scattering and thus affect the measurement and localization of sound source [5,6]. Therefore, it is of great significance to carry out research on the propagation of acoustic waves in the flow.

Numerical simulation is an important means for such research. The main method used is direct numerical simulation (DNS) [7,8], which combines acoustic disturbance and the mean flow and then simulates acoustic waves propagation directly by solving the Navier–Stokes equations. However, DNS requires a very fine grid and a small time step, making its computational cost extremely high. In addition, because acoustic disturbance is usually several orders of magnitude smaller than the mean flow, the calculation of the two parts combined smoothens out the effect of acoustic disturbance, resulting in large error. To overcome these shortcomings, methods of solving perturbation equations such as linearized Euler equations (LEE) [9,10,11] or linearized Navier–Stokes equations (LNSE) [12,13] have been proposed to simulate acoustic wave propagation. These methods essentially solve macroscopic equations, which require high-precision schemes to ensure accuracy. Therefore, the numerical simulation of acoustic wave propagation still needs further development.

Over the last few decades, the lattice Boltzmann method (LBM) has become a popular computational fluid dynamics method [14,15,16,17,18]. LBM is based on molecular dynamics theory, which abstracts fluid into a large number of microscopic particles that collide and migrate through discrete grids according to simple motion rules to illustrate the evolution of the flow field; it reveals macroscopic motion characteristics of a fluid using a particle-distribution function. LBM does not entail solving complex differential equations directly; it only requires solving algebraic equations, which make the calculation process simpler. It has been applied to computational aeroacoustics [19,20,21,22]. Studies have shown that LBM has lower dissipation under the same accuracy, and it is easy to carry out parallel calculations [23], which makes the method suitable for large-scale aeroacoustics simulation. However, in acoustic waves propagation simulation, LBM combines the calculation of acoustic disturbance and the mean flow, which can lead inaccuracies. To better simulate the propagation of acoustic disturbance, the linearized lattice Boltzmann method (LLBM) was established [24,25], which divides the distribution function into a mean component and perturbation parts. Based on the moment relationship between the perturbation distribution function and the perturbation macroscopic variables, the linearized lattice Boltzmann equation (LLBE) can be recovered to linearized macroscopic equations through Chapman–Enskog (C-E) expansion analysis and the evolution of the perturbation distribution function is realized using the standard LBM. It should be pointed out that because the standard LBM can only be applied to uniform grids; special methods are required if it is applied to nonuniform grids. At the same time, it stores the particle velocities and the distribution function of all lattice velocity directions at each grid point, which requires a lot of memory. These deficiencies make it difficult for standard LBM or LLBM to simulate acoustic wave propagation. To solve these deficiencies, Shu et al. proposed the lattice Boltzmann flux solver (LBFS) employing the finite volume method to calculate the flux at an interface [26,27,28,29,30]. Zhan et al. further developed a linearized lattice Boltzmann flux solver (LLBFS) suitable for acoustic propagation simulation [31], wherein the solution of the interface satisfies the lattice Boltzmann equation; this is more in line with physical laws, and the calculation load is comparable to the traditional flux scheme. However, LBFS and LLBFS involve two models, the finite volume method (FVM) and the LBM, which are inconvenient for researchers. Chen et al. recently proposed a simplified lattice Boltzmann method (SLBM) [32,33], which approximates the nonequilibrium part of the distribution function by second-order interpolation of the equilibrium distribution function at other locations and times, so that the evolution of the distribution function can be transformed into the evolution of the equilibrium distribution function. SLBM further simplifies the calculation, and, at the same time, the distribution function in the lattice velocity direction of each particle at each grid point does not need to be stored, which makes it less memory-demanding.

In this paper, a simplified linearized lattice Boltzmann method (SLLBM) that combines the advantages of LLBM and SLBM was proposed and used for acoustic wave propagation simulation. Through C-E expansion analysis, the LLBE was recovered to linearized macroscopic equations; this process was divided into a predictor step and a corrector step using the fractional-step calculation technique. Using second-order interpolation approximation of the perturbation equilibrium distribution function at other positions and times to represent the nonequilibrium part of the perturbation distribution function, the evolution of the latter was transformed into the evolution of the former, and the calculation formulas of SLLBM were deduced. SLLBM inherits the advantages of the LLBM, calculating acoustic disturbance and the mean flow separately so macroscopic variables of the mean flow do not affect the calculation of acoustic disturbance. At the same time, in the SLLBM, the perturbation macroscopic variables were directly evolved so that the evolution and storage of the perturbation distribution function were avoided, which implies only the perturbation macroscopic variables instead of the values of perturbation distribution functions along all lattice velocity directions at each grid point needing to be stored and the physical boundary conditions can be directly processed without converting the perturbation distribution function and perturbation macroscopic variables to each other according to the moment relationships. As a result, SLLBM requires less memory and is simpler to operate than the standard LBM. In addition, to simulate the scattering effect of acoustic waves encountering objects, the immersed boundary method (IBM) was introduced into the framework of SLLBM so that the method can simulate the influence of complex geometries.

The remainder of this paper is arranged as follows. In Section 2, theories related to SLLBM are introduced, including the LLBE and its recovered form, the derivation process of SLLBM, the IBM under the framework of SLLBM, boundary conditions, and the computational sequence. In Section 3, several cases are used to validate the feasibility of SLLBM for acoustic wave propagation simulation. Finally, conclusions are drawn in Section 4.

## 2. Methodology

### 2.1. LLBE and C-E Expansion Analysis

For the lattice Boltzmann equation, the density distribution function f can be divided into the steady mean component $\bar{f}$ and the perturbation part $f^{\prime }$, i.e., $f=\bar{f}+f^{\prime }$. Using the perturbation distribution function, the LLBE with the Bhatnagar–Gross–Krook (BGK) approximation is obtained:

(1) $$\frac{\partial f_{\alpha }^{\prime }}{\partial t}+\xi_{\alpha }\cdot \nabla f_{\alpha }^{\prime }=-\frac{1}{\tau }\left(f_{\alpha }^{\prime }-f_{\alpha }^{\prime }{}^{eq}\right)$$

where $\tau =\frac{\upsilon }{c_{s}^{2}\delta_{t}}+\frac{1}{2}$ is the nondimensional relaxation time, which is associated with the kinematic viscosity υ of the fluid, $\xi_{\alpha }$ and $f_{\alpha }^{\prime }$ represent the component of the lattice velocity and the perturbation distribution function $f^{\prime }$ in direction α, respectively; $f_{\alpha }^{\prime }{}^{eq}$ is the perturbation equilibrium distribution function, which is given by [25]:

(2) $$f_{\alpha }^{\prime }{}^{eq}=\frac{\rho^{\prime }}{\bar{\rho }}{\bar{f}}_{\alpha }^{eq}+\bar{\rho }w_{\alpha }\left(\frac{\xi_{\alpha }\cdot u^{\prime }}{c_{s}^{2}}+\frac{\left(\xi_{\alpha }\cdot u^{\prime }\right)\left(\xi_{\alpha }\cdot \bar{u}\right)}{c_{s}^{4}}-\frac{u^{\prime }\cdot \bar{u}}{c_{s}^{2}}\right)$$

where $c_{s}=1/\sqrt{3}$ is the speed of sound, $w_{\alpha }$ is the weight coefficient of the lattice in direction α; $\bar{\rho }$, $\bar{u}$, and $\rho^{\prime }$, $u^{\prime }$ denote the macroscopic variables, which are divided into the mean flow and acoustic disturbance, respectively; ${\bar{f}}_{\alpha }^{eq}$ is the steady equilibrium distribution function:

(3) $${\bar{f}}_{\alpha }^{eq}=\bar{\rho }w_{\alpha }\left(1+\frac{\xi_{\alpha }\cdot \bar{u}}{c_{s}^{2}}+\frac{{\left(\xi_{\alpha }\cdot \bar{u}\right)}^{2}}{2c_{s}^{4}}-\frac{{\left|\bar{u}\right|}^{2}}{2c_{s}^{2}}\right)$$

The linearized macroscopic variables $\bar{\rho }$, $\bar{u}$, and $\rho^{\prime }$, $u^{\prime }$ and mesoscopic variables have the following moment relationship:

(4) $$\rho^{\prime }=\sum_{\alpha }f_{\alpha }^{\prime }{}^{eq}$$

(5) $$\bar{\rho }u^{\prime }+\rho^{\prime }\bar{u}=\sum_{\alpha }\xi_{\alpha }f_{\alpha }^{\prime }{}^{eq}$$

For two-dimensional problems, the LBM adopts the D2Q9 model; the lattice velocity $\xi_{\alpha }$ and weight coefficient $w_{\alpha }$ are given by:

(6) $$\begin{array}{l} \begin{array}{cc} \begin{array}{cc} \left|\xi_{0}\right|=0, & \left|\xi_{1-4}\right|=1, \end{array} & \left|\xi_{5-8}\right|=\sqrt{2} \end{array}\\ \begin{array}{ccc} w_{0}=\frac{4}{9}, & w_{1-4}=\frac{1}{9}, & w_{5-8}=\frac{1}{36} \end{array} \end{array}$$

C-E expansion analysis is often used to link the kinetic theory of gases and the macroscopic equations of motion [24]. It can also be used to link LLBE and LNSE. By C-E expansion analysis, the perturbation distribution function, time derivative, and spatial derivative can be expanded into the following forms, respectively:

(7) $$f_{\alpha }^{\prime }=f_{\alpha }^{\prime }{}^{\left(0\right)}+Knf_{\alpha }^{\prime }{}^{\left(1\right)}+Kn^{2}f_{\alpha }^{\prime }{}^{\left(2\right)}$$

(8) $$\frac{\partial }{\partial t}=Kn\frac{\partial }{\partial t_{0}}+Kn^{2}\frac{\partial }{\partial t_{1}}$$

(9) $$\nabla =Kn\cdot \nabla_{1}$$

where Kn is the Knudsen number.

By substituting Equations (7)–(9) into the Taylor expansion of LLBE (Equation (1)), the decomposition forms of different orders can be obtained:

(10) $$O\left(Kn^{0}\right):f_{\alpha }^{\prime }{}^{\left(0\right)}=f_{\alpha }^{\prime }{}^{eq}$$

(11) $$O\left(Kn^{1}\right):\left(\frac{\partial }{\partial t_{0}}+\xi_{\alpha }\cdot \frac{\partial }{\partial r_{1}}\right)f_{\alpha }^{\prime }{}^{\left(0\right)}+\frac{1}{\tau }f_{\alpha }^{\prime }{}^{\left(1\right)}=0$$

(12) $$O\left(Kn^{2}\right):\frac{\partial f_{\alpha }^{\prime }{}^{\left(0\right)}}{\partial t_{1}}+\left(1-\frac{1}{2\tau }\right)\left(\frac{\partial }{\partial t_{0}}+\xi_{\alpha }\cdot \nabla_{1}\right)f_{\alpha }^{\prime }{}^{\left(1\right)}+\frac{1}{\tau \delta_{t}}f_{\alpha }^{\prime }{}^{\left(2\right)}=0$$

Sum the zero-order moments and first-order moments of Equations (11) and (12) under the $O\left(Kn^{1}\right)$ and $O\left(Kn^{2}\right)$ orders in all lattice velocity directions and multiply the results with Kn and $Kn^{2}$, respectively; by adding the results separately, we obtain the governing equations of the LLBE recovered by C-E expansion analysis:

(13) $$\frac{\partial \rho^{\prime }}{\partial t}+\nabla \cdot \left(\sum_{\alpha }\xi_{\alpha }f_{\alpha }^{\prime }{}^{eq}\right)=0$$

(14) $$\frac{\partial \left(\rho^{\prime }\bar{u}+\bar{\rho }u^{\prime }\right)}{\partial t}+\nabla \cdot \left(\sum_{\alpha }\xi_{\alpha }^{i}\xi_{\alpha }^{j}f_{\alpha }^{\prime }{}^{eq}+\left(1-\frac{1}{2\tau }\right)\sum_{\alpha }\xi_{\alpha }^{i}\xi_{\alpha }^{j}f_{\alpha }^{\prime }{}^{neq}\right)=0$$

where $f_{\alpha }^{\prime }{}^{neq}=Knf_{\alpha }^{\prime }{}^{\left(1\right)}=-\tau \delta_{t}Df_{\alpha }^{\prime }{}^{eq}$ denotes the perturbation non-equilibrium distribution function, and it satisfies the following moment relationship:

(15) $$\sum_{\alpha }f_{\alpha }^{\prime }{}^{neq}=0,\begin{array}{cc} & \sum_{\alpha }\xi_{\alpha }f_{\alpha }^{\prime }{}^{neq}=0 \end{array}$$

In addition, to restore Equations (13) and (14) to LNSE, the mesoscopic and macroscopic variables need to satisfy the following moment relationship in addition to Equations (4) and (5):

(16) $$\sum_{\alpha }\xi_{\alpha }^{i}\xi_{\alpha }^{j}f_{\alpha }^{\prime }{}^{eq}=\rho^{\prime }c_{s}^{2}\delta_{i,j}+\bar{\rho }u^{\prime }\bar{u}+\rho^{\prime }\bar{u}\bar{u}+\bar{\rho }\bar{u}u^{\prime }$$

(17) $$\sum_{\alpha }\xi_{\alpha }^{i}\xi_{\alpha }^{j}f_{\alpha }^{\prime }{}^{neq}=-\bar{\mu }\left(\nabla u^{\prime }+{\left(\nabla u^{\prime }\right)}^{T}\right)-\mu^{\prime }\left(\nabla \bar{u}+{\left(\nabla \bar{u}\right)}^{T}\right)$$

where $\mu =\bar{\mu }+\mu^{\prime }=\tau \delta_{t}\left(\bar{\rho }+\rho^{\prime }\right)c_{s}^{2}$ is the dynamic viscosity of the fluid.

### 2.2. SLLBM

According to the fractional-step calculation technique, Equations (13) and (14) can be decomposed into two steps: a predictor step and a corrector step:

The predictor step is formulated as follows:

(18) $$\frac{\partial \rho^{\prime }}{\partial t}+\nabla \cdot \left(\sum_{\alpha }\xi_{\alpha }f_{\alpha }^{\prime }{}^{eq}\right)=0$$

(19) $$\frac{\partial \left(\rho^{\prime }\bar{u}+\bar{\rho }u^{\prime }\right)}{\partial t}+\nabla \cdot \left(\sum_{\alpha }\xi_{\alpha }^{i}\xi_{\alpha }^{j}f_{\alpha }^{\prime }{}^{eq}+\frac{1}{2\tau }\sum_{\alpha }\xi_{\alpha }^{i}\xi_{\alpha }^{j}f_{\alpha }^{\prime }{}^{neq}\right)=0$$

The corrector step is formulated as follows:

(20) $$\frac{\partial \rho^{\prime }}{\partial t}=0$$

(21) $$\frac{\partial \left(\rho^{\prime }\bar{u}+\bar{\rho }u^{\prime }\right)}{\partial t}+\nabla \cdot \left(\left(1-\frac{1}{\tau }\right)\sum_{\alpha }\xi_{\alpha }^{i}\xi_{\alpha }^{j}f_{\alpha }^{\prime }{}^{neq}\right)=0$$

In the predictor step, the solution can be advanced using the following relations:

(22) $${\rho^{\prime }}^{*}=\sum_{\alpha }f_{\alpha }^{\prime }{}^{eq}(r-\xi_{\alpha }\delta_{t},t-\delta_{t})$$

(23) $$\bar{\rho }{u^{\prime }}^{*}+{\rho^{\prime }}^{*}\bar{u}=\sum_{\alpha }\xi_{\alpha }f_{\alpha }^{\prime }{}^{eq}(r-\xi_{\alpha }\delta_{t},t-\delta_{t})$$

where $\delta_{t}$ is the time step and ${}^{*}$ is the intermediate value of the perturbation macroscopic variables obtained by solving the predictor step. It can be proven that Equations (22) and (23) can be used to accurately solve Equations (18) and (19).

The Taylor expansion of the perturbation equilibrium distribution function can be obtained by:

(24) $$f_{\alpha }^{\prime }{}^{neq}(r-\xi_{\alpha }\delta_{t},t-\delta_{t})=f_{\alpha }^{\prime }{}^{eq}(r,t)-\delta_{t}Df_{\alpha }^{\prime }{}^{eq}(r,t)-\frac{\delta_{t}}{2\tau }Df_{\alpha }^{\prime }{}^{neq}(r,t)+O(\delta_{t}^{3})$$

By substituting Equation (24) into Equations (22) and (23) and combining the outcome with Equation (15), we obtain:

(25) $$\begin{array}{c} {\rho^{\prime }}^{*}=\sum_{\alpha }f_{\alpha }^{\prime }{}^{eq}(r,t)-\delta_{t}\left[\frac{\partial }{\partial t}\sum_{\alpha }f_{\alpha }^{\prime }{}^{eq}(r,t)+\nabla \cdot \sum_{\alpha }\xi_{\alpha }f_{\alpha }^{\prime }{}^{eq}(r,t)\right]\\ -\frac{\delta_{t}}{2\tau }\left[\frac{\partial }{\partial t}\sum_{\alpha }f_{\alpha }^{\prime }{}^{neq}(r,t)+\nabla \cdot \sum_{\alpha }\xi_{\alpha }f_{\alpha }^{\prime }{}^{neq}(r,t)\right]+O(\delta_{t}^{3}) \end{array}$$

(26) $$\begin{array}{c} \bar{\rho }{u^{\prime }}^{*}+{\rho^{\prime }}^{*}\bar{u}=\sum_{\alpha }\xi_{\alpha }f_{\alpha }^{\prime }{}^{eq}(r,t)-\delta_{t}\left[\frac{\partial }{\partial t}\sum_{\alpha }\xi_{\alpha }f_{\alpha }^{\prime }{}^{eq}(r,t)+\nabla \cdot \sum_{\alpha }\xi_{\alpha }^{i}\xi_{\alpha }^{j}f_{\alpha }^{\prime }{}^{eq}(r,t)\right]\\ -\frac{\delta_{t}}{2\tau }\left[\nabla \cdot \sum_{\alpha }\xi_{\alpha }^{i}\xi_{\alpha }^{j}f_{\alpha }^{\prime }{}^{neq}(r,t)\right]+O(\delta_{t}^{3}) \end{array}$$

According to the moment relationship introduced in Section 1, Equations (25) and (26) are transformed into the following form:

(27) $$\frac{\partial \rho^{\prime }}{\partial t}+\nabla \cdot \left(\sum_{\alpha }\xi_{\alpha }f_{\alpha }^{\prime }{}^{eq}\right)+O(\delta_{t}^{2})=0$$

(28) $$\frac{\partial \left(\bar{\rho }u^{\prime }+\rho^{\prime }\bar{u}\right)}{\partial t}+\nabla \cdot \left[\sum_{\alpha }\xi_{\alpha }^{i}\xi_{\alpha }^{j}f_{\alpha }^{\prime }{}^{eq}(r,t)+\frac{1}{2\tau }\sum_{\alpha }\xi_{\alpha }^{i}\xi_{\alpha }^{j}f_{\alpha }^{\prime }{}^{neq}\right]+O(\delta_{t}^{2})=0$$

where $O(\delta_{t}^{2})$ is a second-order small parameter, which can be ignored. Thus, Equations (27) and (28) can accurately recover the predictor step Equations (18) and (19).

For the linear continuous equation (Equation (13)), the predictor step can be used directly to solve without correction, i.e., ${\rho^{\prime }}^{n+1}=\rho^{*}$, where the superscript ${}^{n+1}$ represents the perturbation macroscopic variables at the next time step. However, for the linear momentum equation (Equation (14)), there is still a deviation $\nabla \cdot \left[\left(1-\frac{1}{\tau }\right)\sum_{\alpha }\xi_{\alpha }^{i}\xi_{\alpha }^{j}f_{\alpha }^{\prime }{}^{neq}\right]$ between Equation (28) and Equation (14), i.e.,:

(29) $$\bar{\rho }{u^{\prime }}^{n+1}+{\rho^{\prime }}^{n+1}\bar{u}=\bar{\rho }{u^{\prime }}^{*}+{\rho^{\prime }}^{*}\bar{u}-\nabla \cdot \left[\left(1-\frac{1}{\tau }\right)\sum_{\alpha }\xi_{\alpha }^{i}\xi_{\alpha }^{j}f_{\alpha }^{\prime }{}^{neq}\right]$$

To calculate Equation (29), similar to the derivation of the predictor step, we can apply Equation (15) into the Taylor expansion of the perturbation nonequilibrium distribution function $f_{\alpha }^{\prime }{}^{neq}(r-\xi_{\alpha }\delta_{t},t)$, and the following relationship can be deduced:

(30) $$-\nabla \cdot \left[\left(1-\frac{1}{\tau }\right)\sum_{\alpha }\xi_{\alpha }^{i}\xi_{\alpha }^{j}f_{\alpha }^{\prime }{}^{neq}(r,t)\right]=\left(1-\frac{1}{\tau }\right)\frac{1}{\delta_{t}}\sum_{\alpha }\xi_{\alpha }f_{\alpha }^{\prime }{}^{neq}(r-\xi_{\alpha }\delta_{t},t)$$

By using Equation (30), Equation (29) can be written as:

(31) $$\bar{\rho }{u^{\prime }}^{n+1}+{\rho^{\prime }}^{n+1}\bar{u}=\bar{\rho }{u^{\prime }}^{*}+{\rho^{\prime }}^{*}\bar{u}+\left(1-\frac{1}{\tau }\right)\sum_{\alpha }\xi_{\alpha }f_{\alpha }^{\prime }{}^{neq}(r-\xi_{\alpha }\delta_{t},t)$$

We therefore obtained simplified calculation formulas for LLBM, which are summarized as follows:

For the linear continuous equation, the perturbation density at the next time step is directly calculated by:

(32) $${\rho^{\prime }}^{n+1}=\sum_{\alpha }f_{\alpha }^{\prime }{}^{eq}(r-\xi_{\alpha }\delta_{t},t-\delta_{t})$$

For the linear momentum equation, the perturbation velocity at the next time step is obtained through the predictor–corrector process as shown below:

It is obtained in the predictor step as follows:

(33) $$\bar{\rho }{u^{\prime }}^{*}+{\rho^{\prime }}^{*}\bar{u}=\sum_{\alpha }\xi_{\alpha }f_{\alpha }^{\prime }{}^{eq}(r-\xi_{\alpha }\delta_{t},t-\delta_{t})$$

It is obtained in the corrector step as follows:

(34) $${u^{\prime }}^{n+1}=\left(\bar{\rho }{u^{\prime }}^{*}+{\rho^{\prime }}^{*}\bar{u}+\left(1-\frac{1}{\tau }\right)\sum_{\alpha }\xi_{\alpha }f_{\alpha }^{\prime }{}^{neq}(r-\xi_{\alpha }\delta_{t},t)-{\rho^{\prime }}^{n+1}\bar{u}\right)/\bar{\rho }$$

The perturbation nonequilibrium distribution function $f_{\alpha }^{\prime }{}^{neq}$ is given by:

(35) $$f_{\alpha }^{\prime }{}^{neq}(r,t)=-\tau \delta_{t}Df_{\alpha }^{\prime }{}^{eq}(r,t)=-\tau \left[f_{\alpha }^{\prime }{}^{eq*}\left(r,t\right)-f_{\alpha }^{\prime }{}^{eq}\left(r-\xi_{\alpha }\delta_{t},t-\delta_{t}\right)\right]$$

where $f_{\alpha }^{\prime }{}^{eq*}\left(r,t\right)$ denotes the perturbation equilibrium distribution function calculated by the intermediate value of the linear macroscopic variables.

### 2.3. IBM

The idea of the IBM is to imagine immersion of the solid in the fluid [34,35,36,37,38,39], and the interaction between the fluid and the solid wall is realized by adding a boundary force term to the right side of the linear momentum equation. Through this treatment, the linear momentum equation can be written as:

(36) $$\begin{array}{l} \frac{\partial (\bar{\rho }u^{\prime }+\rho^{\prime }\bar{u})}{\partial t}+\nabla \cdot (\bar{\rho }u^{\prime }\bar{u}+\rho^{\prime }\bar{u}\bar{u}+\bar{\rho }\bar{u}u^{\prime })=\\ -\nabla (\rho^{\prime }c_{s}^{2})+\nabla \cdot \left[\bar{\mu }\left(\nabla u^{\prime }+{\left(\nabla u^{\prime }\right)}^{T}\right)+\mu^{\prime }\left(\nabla \bar{u}+{\left(\nabla \bar{u}\right)}^{T}\right)\right]+f \end{array}$$

(37) $$f\left(r,t\right)=\int_{\Gamma }F\left(s,t\right)\delta \left(r-R\left(s,t\right)\right)ds$$

where r and R represent the positions of the Euler point and the Lagrangian point, f and F represent the boundary force terms of the Euler point and the Lagrangian point, δ is the Dirac delta function, and s is the index of the Lagrangian point.

The key to wall boundary processing is to solve the boundary force term of the Lagrangian point. In this paper, the method of Chen et al. [37] was used to revise the perturbation velocity ${u^{\prime }}^{n+1}$. In the following derivation process, the revised result of the perturbation velocity ${u^{\prime }}^{n+1}$ is recorded as $u_{I}^{\prime }{}^{n+1}$, which can be evaluated by:

(38) $$u_{I}^{\prime }{}^{n+1}={u^{\prime }}^{n+1}+\Delta u^{\prime }$$

where $\Delta u^{\prime }$ denotes the revise of ${u^{\prime }}^{n+1}$.

The boundary force term f of the Euler point in Equation (36) can be related to $\Delta u^{\prime }$ according to the following formula:

(39) $$f={\rho^{\prime }}^{n+1}\frac{\Delta u^{\prime }}{\delta_{t}}$$

The no-slip boundary condition was adopted for perturbation velocity on the wall boundary, that is, the perturbation velocity of fluid at the Lagrangian point is the same as the perturbation velocity of the immersed object, which can be written as follows:

(40) $$U_{I}^{\prime }{}^{n+1}\left(R_{l}\right)=U_{B}^{\prime }\left(R_{l}\right)$$

where $U_{I}^{\prime }{}^{n+1}$ and $U_{B}^{\prime }$ represent the perturbation velocity of the fluid and boundary, respectively, and the former is obtained by of the perturbation velocity of the Euler point as follows:

(41) $$\begin{array}{c} U_{I}^{\prime }{}^{n+1}\left(R_{l}\right)=\sum_{e}{u^{\prime }}_{}^{n+1}\left(r_{e}\right)K\left(r_{e}-R_{l}\right)\delta_{e}^{2}\\ l=1,2,\ldots ,N\begin{array}{cc} & e=1,2,\ldots ,M \end{array} \end{array}$$

where N and M represent the number of Lagrangian points and Euler points, respectively; $\delta_{e}$ is the grid scale of the Euler grid; and K is the kernel function related to the positions of Lagrangian points and Euler points, which is defined by:

(42) $$K\left(r_{e}-R_{l}\right)=\delta \left(r_{e1}-R_{l1}\right)\delta \left(r_{e2}-R_{l2}\right)$$

where δ is written as:

(43) $$\delta \left(r\right)=\left\{\begin{array}{cc} 1+\frac{\cos\left(\pi \left|r\right|/2\right)}{4} & \left|r\right|\leq 2\\ 0 & \left|r\right|>2 \end{array}\right.$$

In Equation (38), the revised perturbation velocity is obtained by interpolating the perturbation velocity at the Lagrangian point, and the mathematical relationship that satisfies the no-slip boundary condition is as follows:

(44) $$\Delta u^{\prime }\left(r_{e}\right)=\sum_{l}\delta u_{l}^{\prime }K\left(r_{e}-R_{l}\right)\delta_{l}\begin{array}{cc} & l=1,2,\ldots ,N\begin{array}{cc} & e=1,2,\ldots ,M \end{array} \end{array}$$

where $\delta_{l}$ is the scale of the Lagrangian grid.

Combining Equations (38)–(44), a linear system for solving the correction velocity at Lagrangian points can be obtained:

(45) A⋅X=B

where

(46) $$A=\delta_{e}^{2}\left[\begin{array}{cccc} K_{11} & K_{12} & \cdots & K_{1M}\\ K_{21} & K_{22} & \cdots & K_{2M}\\ \vdots & \vdots & \ddots & \vdots \\ K_{N1} & K_{N2} & K_{N3} & K_{NM} \end{array}\right]\cdot \left[\begin{array}{cccc} K_{11} & K_{12} & \cdots & K_{1N}\\ K_{21} & K_{22} & \cdots & K_{2N}\\ \vdots & \vdots & \ddots & \vdots \\ K_{M1} & K_{M2} & K_{M3} & K_{MN} \end{array}\right]$$

(47) $$X={\left[\delta u_{l}^{\prime }{}^{1}\delta_{l}^{1},\delta u_{l}^{\prime }{}^{2}\delta_{l}^{2},\ldots ,\delta u_{l}^{\prime }{}^{N}\delta_{l}^{N}\right]}^{T}$$

(48) $$B=\left[\begin{array}{c} {U^{\prime }}_{B}^{1}\\ {U^{\prime }}_{B}^{2}\\ \vdots \\ {U^{\prime }}_{B}^{N} \end{array}\right]-\left[\begin{array}{cccc} K_{11} & K_{12} & \cdots & K_{1M}\\ K_{21} & K_{22} & \cdots & K_{2M}\\ \vdots & \vdots & \ddots & \vdots \\ K_{N1} & K_{N2} & K_{N3} & K_{NM} \end{array}\right]\left[\begin{array}{c} u_{1}^{{}^{\prime }*}\\ u_{2}^{{}^{\prime }*}\\ \vdots \\ u_{M}^{{}^{\prime }*} \end{array}\right]$$

### 2.4. Boundary Conditions

#### 2.4.1. Periodic Boundary Condition

**Figure 1:**
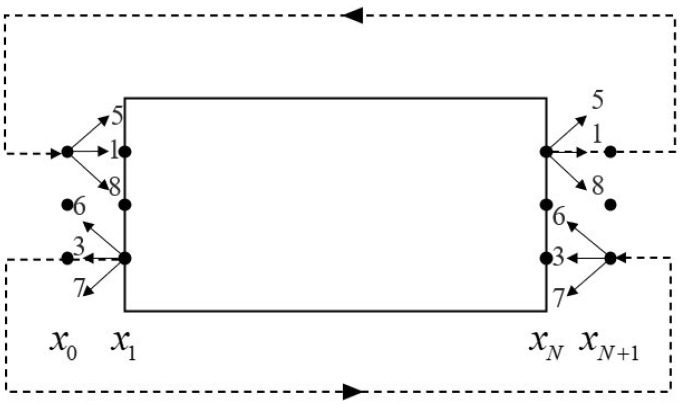
Schematic model of periodic boundary condition.

Here, we adopted the periodic boundary condition [40]. Taking the two-dimensional flow shown in Figure 1 as an example, the fluid flows in from the left and out to the right. There are two layers of virtual grid points $x_{0}$ and $x_{N+1}$ outside the entrance $x_{1}$ on the left and the exit $x_{N}$ on the right, respectively; the periodic boundary conditions are:

(49) $$q_{1,5,8}^{\prime }\left(x_{0},j,t\right)=q_{1,5,8}^{\prime }\left(x_{N},j,t\right)$$

(50) $$q_{3,6,7}^{\prime }\left(x_{N+1},j,t\right)=q_{3,6,7}^{\prime }\left(x_{1},j,t\right)$$

where $q^{\prime }$ represents the perturbation macroscopic variables.

#### 2.4.2. Nonequilibrium Extrapolation Boundary

To process the perturbation nonequilibrium distribution function at the boundary, this paper adopts a nonequilibrium extrapolation boundary condition, which is obtained by interpolating two grid points inside the boundary:

(51) $$f_{i}^{\prime }{}^{neq}\left(x_{0}\right)=f_{i}^{\prime }{}^{neq}\left(x_{1}\right)+\left[f_{i}^{\prime }{}^{neq}\left(x_{1}\right)-f_{i}^{\prime }{}^{neq}\left(x_{2}\right)\right]\frac{x_{i0}-x_{i1}}{x_{i1}-x_{i2}}$$

where $x_{0}$, $x_{1}$, and $x_{2}$ represent the boundary points and the grid points of the first layer and the second layer adjacent to the boundary, respectively. Because the calculation adopts a uniform grid, Equation (51) can be expressed as:

(52) $$f_{i}^{\prime }{}^{neq}\left(x_{0}\right)=2f_{i}^{\prime }{}^{neq}\left(x_{1}\right)-f_{i}^{\prime }{}^{neq}\left(x_{2}\right)$$

### 2.5. Computational Sequence

The computational steps of the SLLBM can be summarized as follows:

(1) Determine the mesh size parameters $\delta_{x}$ and the time step $\delta_{t}$ and then calculate the relaxation time τ.
(2) Calculate the predictor step of the linear governing equations by Equation (33) and obtain the intermediate value of the perturbation macroscopic variables ${q^{\prime }}^{*}$ of the new time step.
(3) According to Equation (35), calculate the perturbation nonequilibrium distribution function $f_{\alpha }^{\prime }{}^{neq}$, selecting appropriate boundary conditions for $f_{\alpha }^{\prime }{}^{neq}$.
(4) Use Equation (34) to calculate the corrector step of the linear momentum equation, and obtain the perturbation velocity ${u^{\prime }}^{n+1}$ of the next time step.
(5) Implement appropriate boundary conditions for the perturbation macroscopic variables and repeat the above process until the results convergent.

For the sound propagation problem that needs to calculate the interaction between the fluid and the solid wall in the fluid, it is necessary to use the IBM derived in Section 1. In this case, the perturbation velocity needs to be revised after step 5. The specific process is as follows:

(1) Solve Equation (45) to obtain the perturbation velocity revision term at the Lagrangian point.
(2) According to the perturbation velocity obtained by Equation (34), combined with Equations (38) and (44), the perturbation velocity of the Euler grid point at the next moment $u_{I}^{\prime }{}^{n+1}$ can be obtained.

### 2.6. Memory Cost

As can be seen from the introduction in Section 2.2, in the SLLBM, the perturbation macroscopic variables were directly evolved so that the evolution and storage of the perturbation distribution function were avoided, which implies only the perturbation macroscopic variables, instead of the values of perturbation distribution functions along all lattice velocity directions at each grid point, need to be stored. As a result, SLLBM requires less memory than the standard LBM.

For instance, during the simulation of the acoustic wave propagation in the two-dimensional imcompressible isothermal flow by the D2Q9 model, only six variables including the present values and the intermediate values of perturbation velocity and density need to be stored at each grid point. Compared with the standard LBM, the number of variables to be stored at each grid point was reduced from 9 to 6, implying the SLLBM can theoretically save about 33.3% of memory [32]. In the simulation of the three-dimensional problem by D3Q19 model, the number of variables to be stored at each grid point was reduced from 19 to 8, which means the SLLBM can theoretically save about 57.9% of memory [41].

## 3. Numerical Examples

In this section, some numerical examples are used to verify the correctness of the SLLBM for the simulation of acoustic waves propagation in the fluid; we consider the following scenarios: (1) propagation of a Gaussian pulse, (2) propagation of a time-periodic sound sources, (3) propagation of plane wave, (4) a Gaussian pulse interacting with a solid wall, and (5) a Gaussian pulse scattered by a stationary circular cylinder.

Cases (1), (2), and (3) test the feasibility and accuracy of SLLBM through the simulation of three different sound sources. Case (4) evaluates the feasibility of SLLBM for calculating the acoustic reflections by a solid wall. Case (5) is used to test the feasibility of introducing the IBM into the SLLBM framework to study the interaction between acoustic waves and complex boundaries.

In these examples, the variables are all nondimensionalized, and the nondimensional parameters of density, velocity, and pressure are $\rho_{\infty }$, $c_{\infty }$, and $\rho_{\infty }c_{\infty }^{2}$, respectively.

### 3.1. Case 1: Propagation of a Gaussian Pulse

**Figure 2:**
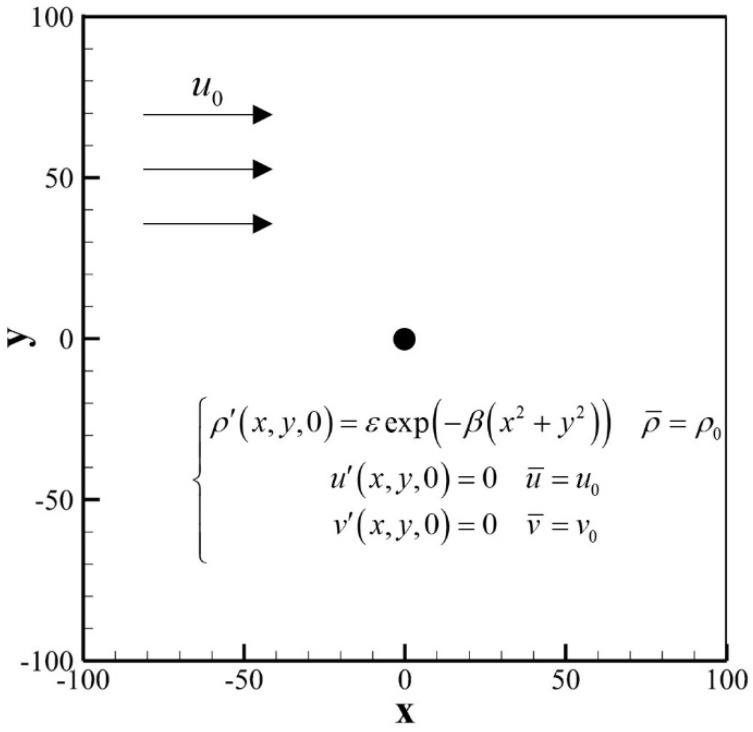
Computational model of a Gaussian pulse propagation.

**Figure 3:**
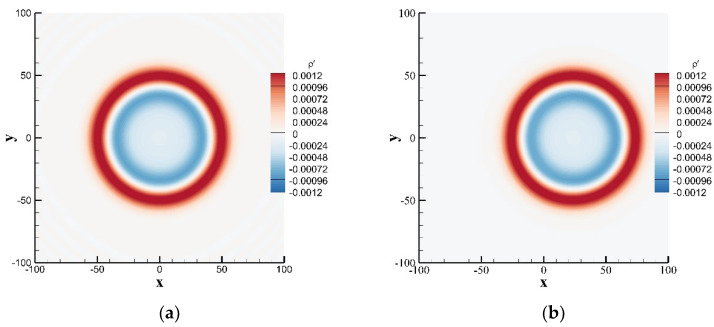
Instantaneous perturbation density contours of a Gaussian pulse obtained by the SLLBM at t=80. (**a**) stationary medium $\left(\bar{u}=0.0\right)$, (**b**) moving medium $\left(\bar{u}=0.3\right)$.

**Figure 4:**
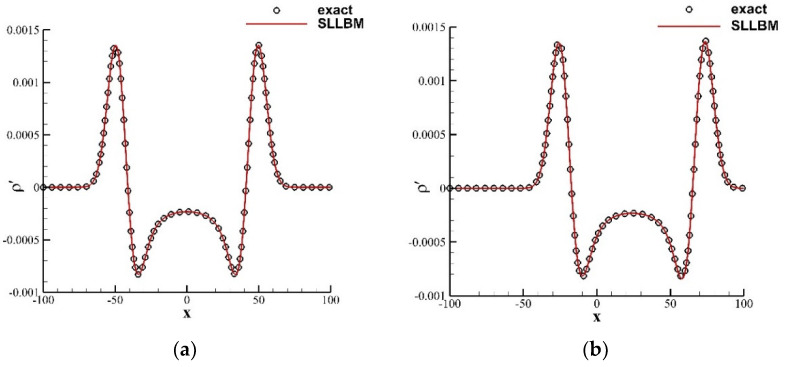
Instantaneous perturbation density distribution along the centerline at y=0 for t=80. (**a**) stationary medium $\left(\bar{u}=0.0\right)$, (**b**) moving medium $\left(\bar{u}=0.3\right)$.

As shown in Figure 2, the computational domain of Gaussian pulse propagation is $\left[-200,200\right]\times \left[-200,200\right]$, the grid points are uniformly arranged, the grid scale $\delta_{x}=1.0$, the time step $\delta_{t}=1.0$, and the relaxation time τ=0.5. At the initial moment, a Gaussian pulse was applied with the following formula:

(53) $$\left\{\begin{array}{c} \begin{array}{cc} \rho^{\prime }\left(x,y,0\right)=\varepsilon \exp\left(-\beta \left(x^{2}+y^{2}\right)\right) & \bar{\rho }=\rho_{0} \end{array}\\ \begin{array}{cc} u^{\prime }\left(x,y,0\right)=0 & \bar{u}=u_{0} \end{array}\\ \begin{array}{cc} v^{\prime }\left(x,y,0\right)=0 & \bar{v}=v_{0} \end{array} \end{array}\right.$$

where $\rho_{0}=1.0$ represents the density of the uniform mean flow, ε=0.01 is the density pulse amplitude; and β is the source shape factor obtained by $\beta =\ln2/b^{2}$, where b=8 representing the half-width Gaussian factor. For this form of Gaussian impulse propagation, the exact solution for the perturbation density $\rho^{\prime }$ is described by [42]:

(54) $$\rho^{\prime }\left(x,y,t\right)=\frac{\varepsilon }{2\beta }\int_{0}^{\infty }\exp\left(-\psi^{2}/4\beta \right)\cos\left(c_{s}t\psi \right)J_{0}\left(\psi \eta \right)\psi d\psi$$

where $\eta ={\left[{\left(x-u_{0}t_{0}\right)}^{2}+y^{2}\right]}^{\frac{1}{2}}$, and $J_{0}\left(\cdot \right)$ is the zero-order Bessel function of the first kind. For both cases of stationary medium $\bar{u}=0.0$ and moving medium $\bar{u}=0.3$, Figure 3 shows the contours of instantaneous perturbation density at t=80, and Figure 4 shows a comparison of the instantaneous perturbation density and the exact solution along the centerline at y=0. The calculation results of the SLLBM are in good agreement with the exact solutions regardless of whether there is the convective effect, which shows that SLLBM can simulate the acoustic waves propagation problems in stationary and moving medium.

### 3.2. Case 2: Propagation of a Time-Periodic Acoustic Source

As the second case, we simulate the propagation of a time-periodic acoustic source in a stationary medium. The acoustic source is given by the following formula:

(55) $$\left\{\begin{array}{c} \begin{array}{cc} \rho^{\prime }\left(x,y,0\right)=\varepsilon \sin\left(\omega t\right) & \bar{\rho }=\rho_{0} \end{array}\\ \begin{array}{cc} u^{\prime }\left(x,y,0\right)=0 & \bar{u}=u_{0} \end{array}\\ \begin{array}{cc} v^{\prime }\left(x,y,0\right)=0 & \bar{v}=v_{0} \end{array} \end{array}\right.$$

where ε=0.01 is the density pulse amplitude, ω=π/10 represents the frequency of the time-periodic acoustic source; and $\left(\rho_{0},u_{0},v_{0}\right)=\left(1.0,0.0,0.0\right)$ are the variables in the stationary flow. The computation domain is a $\left[-50,50\right]\times \left[-50,50\right]$ square, and a uniform grid is used, giving the grid spacing and the time step of 1.0.

**Figure 5:**
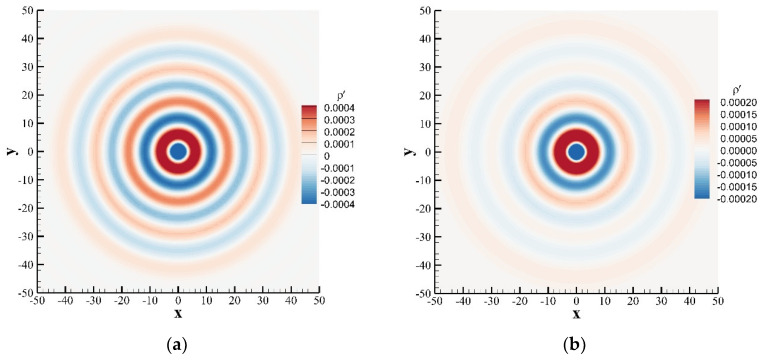
Instantaneous perturbation density contours of a time-periodic acoustic source in a stationary flow obtained by the SLLBM at t=75. (**a**) τ=0.6, (**b**) τ=1.0.

**Figure 6:**
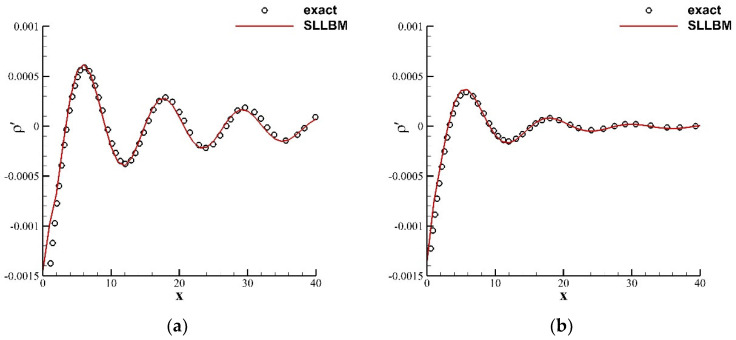
Instantaneous perturbation density distribution along centerline at t=75 for y=0. (**a**) τ=0.6, (**b**) τ=1.0.

For the static medium, Figure 5 shows the instantaneous perturbation density contours of the time-periodic acoustic source in the stationary medium at t=75 for two relaxation times τ=0.6 and 1.0. The SLLBM clearly captures the sound wave generated at the origin and as it propagates outward, and the attenuation speed of the acoustic waves amplitude is significantly greater when τ=1.0. For quantitative analysis, Figure 6 shows a comparison of the instantaneous perturbation density curve along the centerline at t=75 with the exact solution [43]. As can be seen, the results calculated by SLLBM are in good agreement with the exact solution.

**Figure 7:**
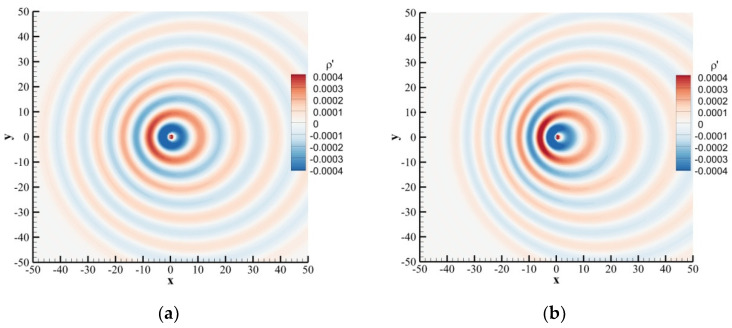
Instantaneous perturbation density contours of a time-periodic acoustic source in a uniform flow obtained by the SLLBM at t=100. (**a**) $\bar{u}=0.1$, (**b**) $\bar{u}=0.2$.

For the moving medium, the Mach number of the uniform flow was set as 0.1 or 0.2, and relaxation time as τ=0.6. Figure 7 shows the perturbation density contours at t=100. It can be seen that the wavelengths were shorter in the left and longer in the right of the sound source because of the Doppler effect. The wavelengths of acoustic waves located on the left and right sides of the sound source should be [43]:

(56) $$\lambda_{left,right}=\left(c_{s}\mp \bar{u}\right)T$$

**Figure 8:**
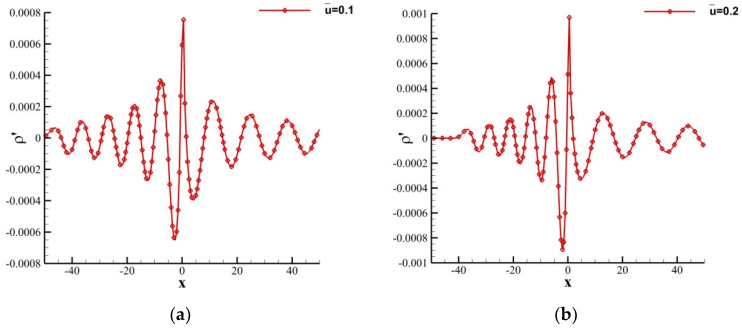
Instantaneous perturbation density distribution along centerline at t=100 for y=0. (**a**) $\bar{u}=0.1$, (**b**) $\bar{u}=0.2$.

Since $T=\frac{2\pi }{\omega }=20$, $c_{s}=0.586$, and $\bar{u}=0.1$ or 0.2, the wavelengths of acoustic waves located on the left sides of the sound source should be 9.72 and 7.72, and the wavelengths on the right side should be 13.72 and 15.72, respectively. For quantitative analysis, the instantaneous perturbation density curves along the centerline at t=100 are shown in Figure 8, from which the Doppler effect is clear. It can be seen that the wavelengths of acoustic waves located on the left sides of the sound source $\lambda_{left}\approx 9.64$ or 7.80, and the wavelengths on the right side $\lambda_{right}\approx 13.57$ or 15.73, which shows that SLLBM can also well simulate the convection effect of the moving medium.

### 3.3. Case 3: Propagation of Plane Wave

**Figure 9:**
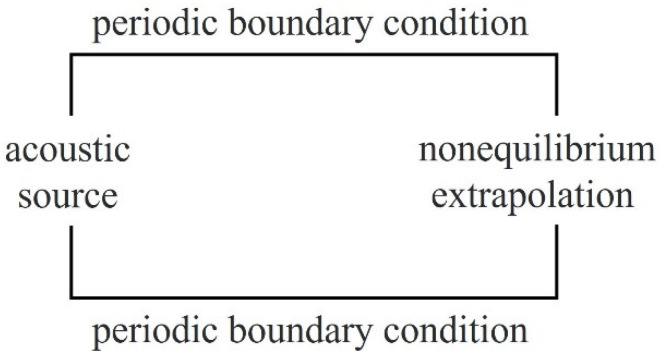
Schematic model of the plane wave calculation model.

In this case, we simulate the propagation of a one-dimensional plane wave. The calculation model is shown in Figure 9. On the top and bottom boundaries, a periodic boundary was applied, the right side is a nonequilibrium extrapolation boundary, and the left side is a sound source, which is given by the following formula:

(57) $$\left\{\begin{array}{c} \begin{array}{cc} \rho^{\prime }\left(x,y,0\right)=\varepsilon \sin\left(\omega t\right) & \bar{\rho }=\rho_{0} \end{array}\\ \begin{array}{cc} u^{\prime }\left(x,y,0\right)=c_{s}\varepsilon \sin\left(\omega t\right)/\bar{\rho } & \bar{u}=u_{0} \end{array}\\ \begin{array}{cc} v^{\prime }\left(x,y,0\right)=0 & \bar{v}=v_{0} \end{array} \end{array}\right.$$

where ε=0.01, $\left(\rho_{0},u_{0},v_{0}\right)=\left(1.0,0.0,0.0\right)$ are the variables in the stationary flow, $\omega =2\pi c_{s}/\lambda$ represents the frequency of the sound source, and λ denotes the wavelength. For this defined one-dimensional plane wave propagation, the exact solution for the perturbation velocity $u^{\prime }$ is given by:

(58) $$u^{\prime }\left(x,t\right)=\frac{c_{s}\varepsilon }{\rho_{0}}e^{-\varphi x}\sin\left(\omega t-kx\right)$$

where $\varphi =4\pi^{2}\upsilon /c_{s}\lambda^{2}$ represents the attenuation coefficient of the acoustic wave. The calculation domain was set at $\left[0,20\right]\times \left[0,1000\right]$, the calculation grid adopts a uniform grid, the grid scale $\delta_{x}=1.0$, and the time step $\delta_{t}=1.0$.

**Figure 10:**
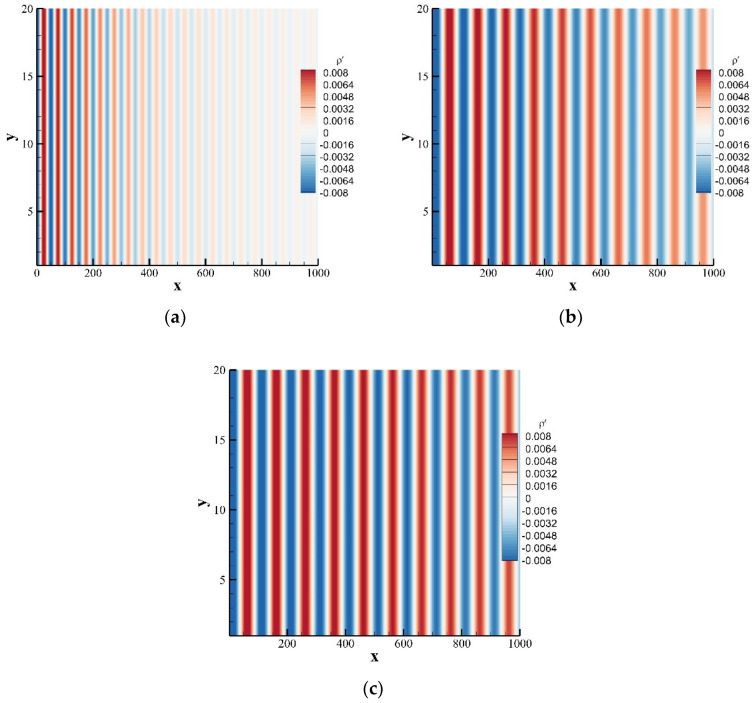
Instantaneous perturbation density contours of plane wave obtained by the SLLBM at t=5000. (**a**) $\left(\upsilon ,\lambda \right)=\left(0.1,50\right)$, (**b**) $\left(\upsilon ,\lambda \right)=\left(0.1,100\right)$, (**c**) $\left(\upsilon ,\lambda \right)=\left(0.05,100\right)$.

**Figure 11:**
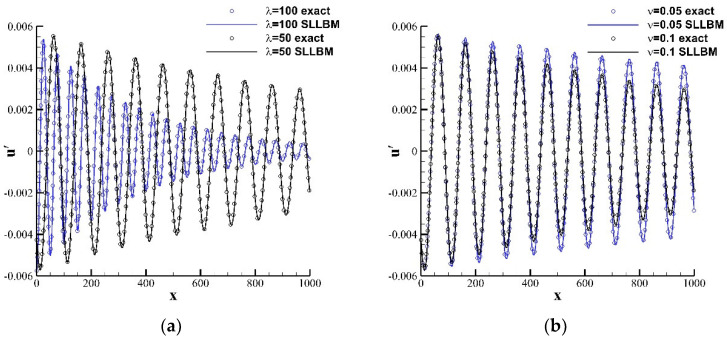
Instantaneous perturbation density distribution along the centerline y=10 at t=5000. (**a**) υ=0.1, (**b**) λ=100.

Figure 10 provides the perturbation density contours for different kinematic viscosities and different wavelengths at t=75. The plane wave propagates in the form of a band, and the larger the wavelength and the smaller the kinematic viscosity, the slower the acoustic waves decays during the propagation process. To specifically judge the influence of wavelength and kinematic viscosity on the propagation of the one-dimensional plane wave, Figure 11 plots the comparison of the instantaneous perturbation velocity $u^{\prime }$ distribution with the exact solution at the position y=10. During the propagation of a one-dimensional plane wave, the wavelength determines the phase of the acoustic wave, and the kinematic viscosity determines the amplitude. For plane wave propagation with different kinematic viscosities or wavelengths, the results obtained by SLLBM are in good agreement with the exact solutions.

### 3.4. Case 4: Propagation of a Gaussian Pulse with Wall Reflection

**Figure 12:**
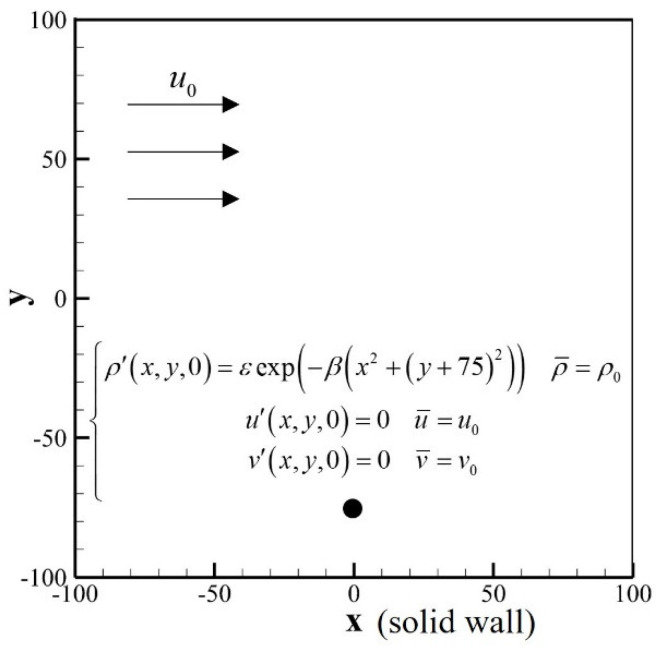
Computational model of Gaussian pulse propagation with wall reflection.

This case simulates the propagation of a Gaussian pulse with wall reflections by SLLBM. The calculation model is shown in Figure 12 and the sound source is defined by:

(59) $$\left\{\begin{array}{c} \begin{array}{cc} \rho^{\prime }\left(x,y,0\right)=\varepsilon \exp\left(-\beta \left({\left(x-0\right)}^{2}+{\left(y+75\right)}^{2}\right)\right) & \bar{\rho }=\rho_{0} \end{array}\\ \begin{array}{cc} u^{\prime }\left(x,y,0\right)=0 & \bar{u}=u_{0} \end{array}\\ \begin{array}{cc} v^{\prime }\left(x,y,0\right)=0 & \bar{v}=v_{0} \end{array} \end{array}\right.$$

where ε=0.01, $\left(\rho_{0},u_{0},v_{0}\right)=\left(1.0,0.0,0.0\right)$ are the variables in the stationary flow and $\beta =\ln2/3^{2}$. The calculation domain was set at $\left[-100,100\right]\times \left[-100,100\right]$, the calculation adopts a uniform grid, the grid scale $\delta_{x}=0.5$, and the time step $\delta_{t}=0.5$, and the relaxation time τ=0.5. The exact solution for the perturbation density $\rho^{\prime }$ is defined by:

(60) $$\rho^{\prime }\left(x,y,t\right)=\frac{\varepsilon }{\beta }\int_{0}^{\infty }\exp\left(-\psi^{2}/4\beta \right)\cos\left(c_{s}t\psi \right)\left[J_{0}\left(\psi \eta_{1}\right)+J_{0}\left(\psi \eta_{2}\right)\right]\psi d\psi$$

where $\eta_{1}={\left[{\left(x-u_{0}t\right)}^{2}+{\left(y+75\right)}^{2}\right]}^{0.5}$, $\eta_{2}={\left[{\left(x-u_{0}t\right)}^{2}+{\left(y+125\right)}^{2}\right]}^{0.5}$.

**Figure 13:**
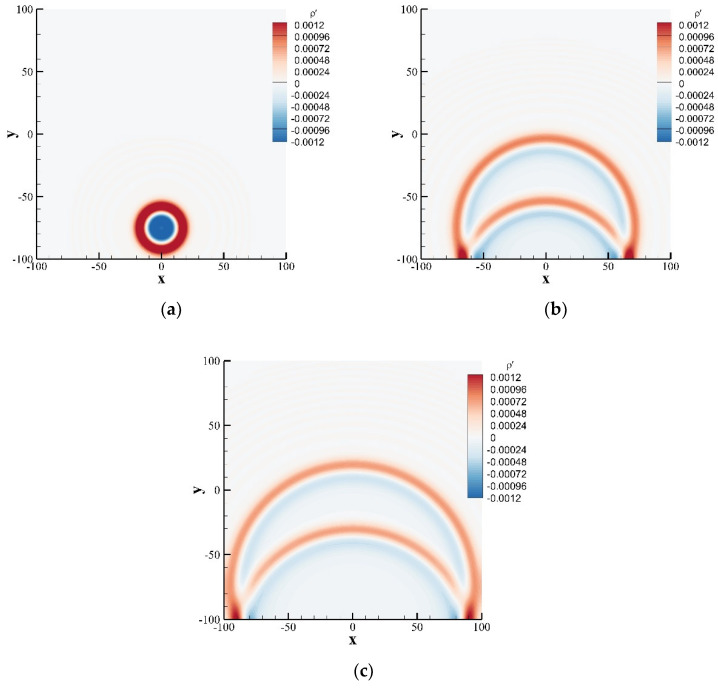
Instantaneous perturbation density contours of a Gaussian pulse with wall reflection obtained by the SLLBM. (**a**) t=26, (**b**) t=120, (**c**) t=160.

**Figure 14:**
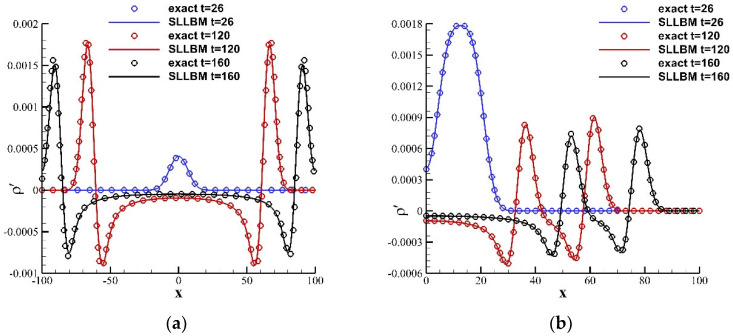
Instantaneous perturbation density distribution at different locations at $t=\begin{array}{ccc} 26, & 120, & 160 \end{array}$. (**a**) y=−100, (**b**) x=y+100.

Figure 13 shows the perturbation density contours at t= 26, 120, and 160 obtained by the SLLBM. Figure 14 shows the perturbation density distributions along the reflecting wall y=−100 and x=y+100 at these three moments calculated by the SLLBM and compared with the exact solution. There are two peaks along x=y+100 at t=120, 160, the inner peak is generated by the reflection of the pulse with the wall, and the outer one is generated by the propagation of the pulse. The numerical solutions are in good agreement with the exact solution, which shows that SLLBM can simulate the problem of acoustic waves encountering wall reflections.

### 3.5. Case 5: A Gaussian Pulse Scattered by a Stationary Cylinder

**Figure 15:**
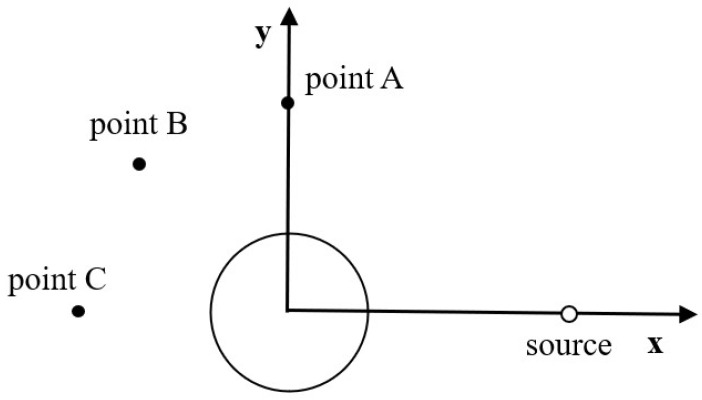
Computational model of a Gaussian pulse scattered by a stationary circular cylinder.

**Figure 16:**
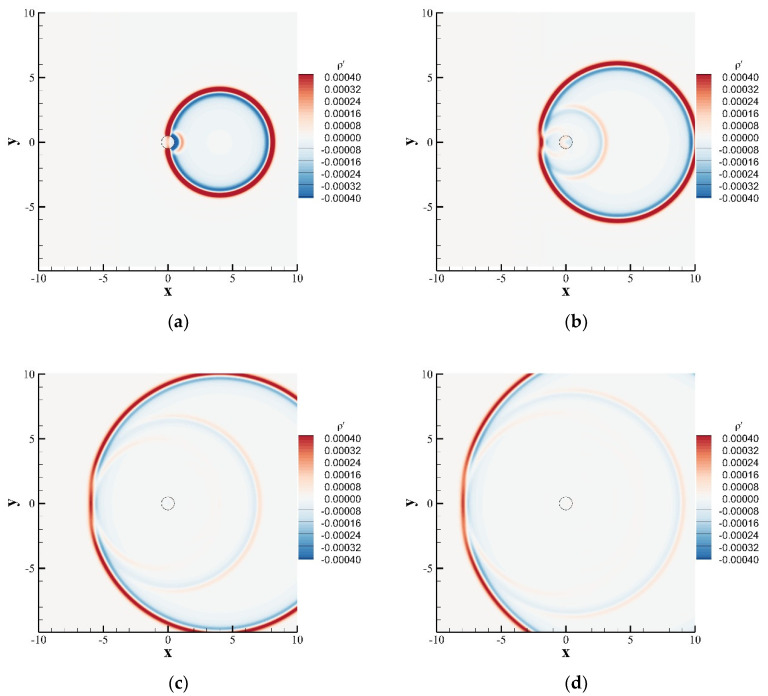
Disturbance density contours of Gaussian pulse scattering with a circular cylinder obtained by the SLLBM-IBM. (**a**) $tc_{s}=4$, (**b**) $tc_{s}=6$, (**c**) $tc_{s}=10$, (**d**) $tc_{s}=12$.

**Figure 17:**
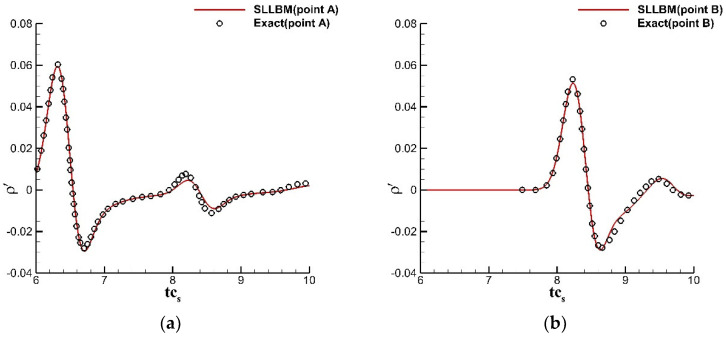

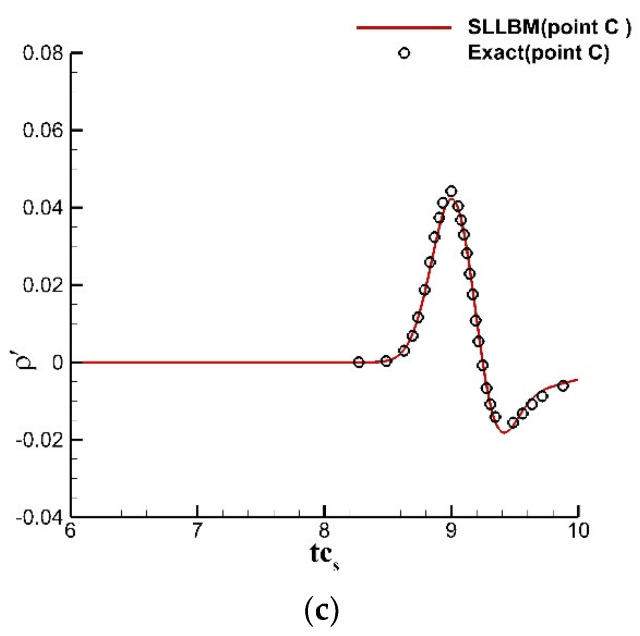
Disturbance density curves of a Gaussian pulse scattering with a circular cylinder obtained by the SLLBM-IBM. (**a**) point A, (**b**) point B, (**c**) point C.

In this problem, a stationary circular cylinder (radius *R* = 10) is located at the origin. At the initial moment, the sound source is applied as follows (Figure 15):

(61) $$\left\{\begin{array}{c} \begin{array}{cc} \rho^{\prime }\left(x,y,0\right)=\varepsilon \exp\left(-\beta \left({\left(x-400.0\right)}^{2}+{\left(y-0.0\right)}^{2}\right)\right), & \bar{\rho }=\rho_{0} \end{array}\\ \begin{array}{cc} u^{\prime }\left(x,y,0\right)=0 & \bar{u}=u_{0} \end{array}\\ \begin{array}{cc} v^{\prime }\left(x,y,0\right)=0 & \bar{v}=v_{0} \end{array} \end{array}\right.$$

where ε=0.01, $\left(\rho_{0},u_{0},v_{0}\right)=\left(1.0,0.0,0.0\right)$, and β=ln2. The calculation grid adopts a uniform grid, the grid scale $\delta_{x}=1.0$, and the time step $\delta_{t}=1.0$. The circular cylinder was treated using the immersion boundary method, the surface is described by 150 uniform Lagrangian points, and the far-field was treated using the nonequilibrium extrapolation method. Three monitoring points A, B, and C are located at $\left(0,5\right)$, $\left(5\cos\left(3\pi /4\right),5\sin\left(3\pi /4\right)\right)$, $\left(-5,0\right)$ in the computational domain. Figure 16 shows the instantaneous density contours at $tc_{s}=4$, $tc_{s}=6$, $tc_{s}=10$, and $tc_{s}=12$. The propagation of the pulse wave and the interaction with the circular cylinder are shown. Figure 17 shows a comparison of the perturbation density at the three monitoring points with the exact solution.The numerical solution calculated by SLLBM-IBM is in good agreement with the exact solution [44], which quantitatively verifies the correctness of this method.

## 4. Conclusions

SLLBM was proposed and applied to the simulation of acoustic wave propagation in fluids. This method recovered the LLBE to LNSE by C-E expansion analysis, and adopted the fractional-step calculation technique; the predictor-corrector formula of SLLBM was derived. Because the perturbation nonequilibrium distribution function can be approximated by second-order interpolation of the perturbation equilibrium distribution function at other positions and times, the evolution of the perturbation distribution function could be transformed into the evolution of the perturbation equilibrium distribution function. Compared with standard LBM, SLLBM calculates the acoustic disturbance and the mean flow separately, so macroscopic variables of the mean flow do not affect the calculation of acoustic disturbance. At the same time, SLLBM has other advantages: the calculation process is simpler, and the cost of computing memory is reduced. In addition, to simulate the scattering effect of acoustic waves encountering objects, the immersed boundary method (IBM) is within the framework of SLLBM so that the method can simulate the influence of complex geometries.

Various numerical cases, including the propagation of a Gaussian pulse and the interaction with a wall or cylinder, the propagation of a time-periodic acoustic source, and plane wave, were simulated to validate the accuracy of SLLBM. The results obtained by SLLBM are in good agreement with the exact solutions, which proves the accuracy and feasibility of SLLBM in the simulation of acoustic wave propagation.

## Data Availability

Not applicable.

## Abbreviations

$c_{s}$	speed of sound	ξ	lattice velocity
f	density distribution function	w	weight coefficient of the lattice
f	boundary force terms of the Euler point	ρ	denisity
F	boundary force terms of the Lagrangian point	υ	kinematic viscosity
K	kernel function	μ	dynamic viscosity
Kn	Knudsen number	τ	relaxation time
M	number of the Euler points		
N	number of the Lagrangian point	Superscripts	
r	position of the Euler point	’	perturbation part
s	index of the Lagrangian point	$\bar{}$	steady mean part
u	velocity vector	eq	equilibrium distribution function
$U_{I}$	velocity of the fluid	neq	non-equilibrium distribution function
$U_{B}$	velocity of the immersed boundary	*	intermediate variables
R	position of the Lagrangian point	n+1	variables at the next time step
$\delta_{t}$	time step		
$\delta_{x}$	lattice spacing	Subscripts	
$\delta_{e}$	scale of the Euler grid	α	direction
$\delta_{l}$	scale of the Lagrangian grid	e	Euler grid point
λ	wavelength	l	Lagrangian grid point
